# Investigating the shared genetic architecture between obesity and depression: a large-scale genomewide cross-trait analysis

**DOI:** 10.3389/fendo.2025.1578944

**Published:** 2025-05-08

**Authors:** Lei Yuan, Yale Su, Jiangqi Zhao, Minkyoung Cho, Duo Wang, Long Yuan, Mixia Li, Dongdong Zheng, Hulin Piao, Yong Wang, Zhicheng Zhu, Dan Li, Tiance Wang, Ki-Tae Ha, Wonyoung Park, Kexiang Liu

**Affiliations:** ^1^ Department of Cardiovascular Surgery, The Second Hospital of Jilin University, Changchun, China; ^2^ Department of Dermatology, The Second Hospital of Jilin University, Changchun, China; ^3^ Department of Parasitology and Tropical Medicine, and Institute of Health Sciences, Gyeongsang National University College of Medicine, Jinju, Republic of Korea; ^4^ School of Acupuncture, Moxibustion and Tuina, The Third Clinical Medical School of Henan University of Chinese Medicine, Zhengzhou, China; ^5^ Department of Korean Medical Science, School of Korean Medicine, Pusan National University, Yangsan, Republic of Korea; ^6^ Korean Medical Research Center for Healthy Aging, Pusan National University, Yangsan, Republic of Korea

**Keywords:** obesity, depression, genome-wide association study, SCG3, FLRT2

## Abstract

**Introduction:**

Increasing evidence suggests that individuals with obesity are at a higher risk of developing depression, and conversely, depression can contribute to the onset of obesity, creating a detrimental cycle. This study aims to investigate the potential shared biological pathways between obesity and depression by examining genetic correlations, identifying common polymorphisms, and conducting cross-trait genetic analyses.

**Methods:**

We assessed the genetic correlation between obesity and depression using linkage disequilibrium score regression and high-density lipoprotein levels. We combined two different sources of obesity data using METAL and employed bidirectional Mendelian randomization to determine the causal relationship between obesity and depression. Additionally, we conducted multivariate trait analysis using the MTAG method to improve statistical robustness and identify novel genetic associations. Furthermore, we performed a thorough investigation of independent risk loci using GCTA-COJO, PLACO, MAGMA, POPS, and SMR, integrating different QTL information and methods to further identify risk genes and proteins.

**Results:**

Our analysis revealed genetic correlations and bidirectional positive causal relationships between obesity and depression, highlighting shared risk SNP (rs10789340). We identified RPL31P12, NEGR1, and DCC as common risk genes for obesity and depression. Using the BLISS method, we identified SCG3 and FLRT2 as potential drug targets.

**Limitation:**

Most of our data sources are from Europe, which may limit the generalization of our findings to other ethnic populations.

**Conclusion:**

This study demonstrates the genetic causal relationship and common risk SNPs, genes, proteins, and pathways between obesity and depression. These findings contribute to a deeper understanding of their pathogenesis and the identification of potential therapeutic targets.

## 1 Introduction

The Depression and obesity are prevalent conditions with profound public health consequences. In the United States, the lifetime prevalence of major depressive disorder approaches 20% (1–3). Extensive epidemiological research and meta-analyses have established that these conditions frequently co-occur (4). A recent study demonstrated that individuals with obesity are 55% more likely to experience lifetime depression compared to the general population, while those suffering from depression are 58% more likely to become obese (5). This reciprocal relationship is consistently observed across various studies, including a meta-analysis of 17 community-based cross-sectional studies, which revealed a positive correlation between depression and obesity, indicating an 18% increased risk of obesity among those with depression (6–8). Further, substantial evidence suggests that depression and obesity engage in a cyclical relationship, exacerbating one another through adverse physiological changes (9, 10). These include dysregulation of the hypothalamic-pituitary-adrenal axis, heightened inflammation, increased oxidative stress, and hormonal imbalances (11). Individuals with depression might gain weight over time due to a dysregulated stress response or unhealthy lifestyle choices. Conversely, obesity can lead to depression due to negative self-perception or the physical burdens it imposes (12).

Research focused on a single disease may overlook critical genetic loci and molecular regulatory mechanisms. Therefore, employing multivariate analysis methods is essential to broaden the phenotypic spectrum of research, explore risk loci, and delve into the common genetic causes of diseases (13). Shared genetic causes also suggest potential pleiotropy, which often represents genetic confounding factors in trait associations (14, 15). Cross-trait analysis is thus proposed to investigate pleiotropic genetic variations or loci among multiple traits using the correlation of genome-wide association studies (GWAS) signals (16, 17). These pleiotropic loci could serve as intervention targets, potentially preventing or treating these diseases simultaneously.

**Figure 1:**
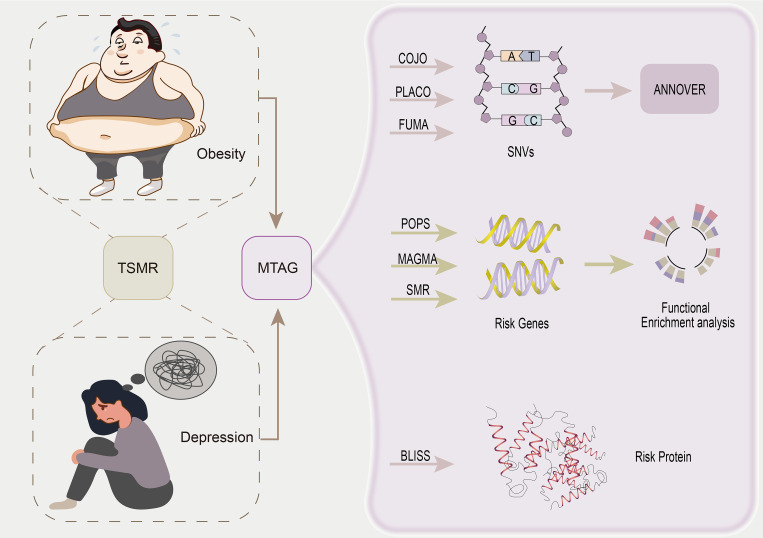
Overall study design.

In this genome-wide pleiotropy association study, we utilized obesity data from Finngen and UKB and depression data from the PGC database. Various statistical genetic methods were employed to study pleiotropic associations sequentially at the single nucleotide variant (SNV), gene, and protein levels, as well as biological pathways, to unravel potential common genetic causes. Firstly, we assessed genetic correlation using Linkage disequilibrium score regression (LDSC) and high-definition likelihood (HDL). Within the framework of pleiotropy analysis, we identified shared pleiotropic genetic loci for obesity and depression at the SNV level using multi-trait analysis of GWASs (MTAG), PLACO, and COJO analyses. Subsequently, we conducted gene-level analyses using MAGMA, POPS, and SMR to identify candidate related risk genes and employed BLISS to identify risk proteins at the protein level. Finally, we performed genome-specific enrichment analyses to characterize genomic pathways and tissue specificity. **Figure 1** illustrates the overall study design.

## 2 Materials and methods

### 2.1 Data collection

#### 2.1.1 Collection of GWAS summary data

A comprehensive range of GWAS summary data was used in this study. Owing to the limited availability of data from other ancestries, we included GWAS summary data from European ancestry. **Supplementary Table S1** presents a comprehensive description of the characteristics of each dataset utilized in our study.

GWAS summary data on obesity were extracted from the UKbiobank, which included 4,688 patients and 458,322 control individuals, and a FinnGen database (Finn-Obesity), which included 23,971 patients and 388,084 control individuals. Depression GWAS data were obtained from the PGC database (246,363 cases and 561,190 controls).To investigate the genetic composition of patients with Obesity, we integrated quantitative trait loci (QTL) data, including expression QTLs (eQTLs) from 54 specific samples (such as gastrointestinal tissues and blood) and plasma protein QTLs (pQTLs). Blood eQTL data were extracted from the expansive eQTLGen Consortium database, which documented single nucleotide polymorphisms (SNPs) associated with traits in 31,684 individuals (18). Plasma pQTL data were extracted from the deCODE database, which included 35,559 Icelandic participants and focused on 4,907 plasma proteins (19). 4953 plasma proteins from the ARIC database, and the UKBPP project, which included 54,219 participants from the UK Biobank study and focused on 2,923 plasma proteins (20).

### 2.2 Statistical analysis

During the analysis phase of this study, we meticulously excluded SNVs within the major histocompatibility complex (MHC) region on chromosome 6 (25–35 Mb) to mitigate potential confounding effects. In addition, we removed SNPs with a minor allele frequency of <0.01 and those with duplicated or missing reference cluster IDs from each GWAS summary dataset for subsequent analysis. Data aligned to the GRCh38 reference were converted to GRCh37 using the liftOver tool for consistency (21).

#### 2.2.1 Assessment of genetic correlations between obesity and depression

LDSC and HDL methods were used to assess the genetic relationship between obesity and depression based on GWAS summary data from two datasets. The HDL method was cross-validated against LDSC to ensure robustness. Significant genetic correlations between obesity and depression were consistently observed across three independent sources, warranting further analysis. To enhance rigor and reliability, multiple comparisons were adjusted using the Bonferroni correction, setting the significance threshold at P < 0.025. Notably, significant genetic correlations were identified between obesity (FinnGen) and depression, as well as obesity (UK Biobank) and depression, highlighting potential shared genetic components.

#### 2.2.2 Meta-analysis of GWASs

Meta-analysis was performed to combine data from the two datasets (UKbiobank, FinnGen). Given the potential sample overlap between the datasets, Metasoft was used to evaluate heterogeneity (I2) and *P*-values based on Cochran’s Q test (P_het). When heterogeneity was present (I2 ≥ 50 or P_het < 0.05), *P*-values from the random-effect model calculated using RE2C were considered (22, 23).

#### 2.2.3 Multi-trait analysis of GWAS summary statistics

Building on the results of our previous phase of research, we continued with a MTAG method, cross-analyzing the META-OBESITY and depression phenotypes. The MTAG method improves the efficiency of identifying associated genes by integrating the genetic correlation structure of several similar traits into a single “meta-analysis,” thereby enabling joint analysis of multiple traits (24). In this analysis, we combined GWAS data for META-OBESITY and depression to derive MTAG-OBESITY. For this purpose, the genome-wide significance threshold was set at a P-value of less than 5e-8 to ensure stringent identification of associations.

We applied PLACO to conduct a genome-wide search for SNPs influencing the risk of both MTAG- OBESITY and depression. In brief, PLACO is a novel statistical approach that identifies pleiotropic loci between two traits by testing the composite null hypothesis (i.e., a locus is associated with zero or one of the traits) (17).

#### 2.2.4 Identification of genetic risk factors for obesity

##### 2.2.4.1 Identification of independent risk loci

To identify genomic risk factors for obesity, we detected distinct independent signals within the genomic loci associated with MTAG-OBESITY using the stepwise model selection framework provided by genome-wide complex trait analysis (GCTA)-conditional and joint analysis (COJO) (25, 26). Based on the results of PLACO, the identified pleiotropic loci were mapped to neighboring genes to investigate their shared biological mechanisms. The functional mapping and annotation (FUMA) platform (27) was used to delineate the genomic risk loci through functional annotation of the variants based on LD scores obtained from the European cohort of the third phase of the 1000 Genomes Project. SNV, validated by COJO and FUMA analyses, was identified as a risk factor for obesity and depression co-morbidity. These variants were annotated using ANNOVAR (28), and their potential deleterious effects were assessed using Combined Annotation Dependent Depletion (CADD) scores, with values of >12.37 indicating a higher likelihood of producing deleterious effects (29).

##### 2.2.4.2 Genetic insights into obesity-depression comorbidity

In the integrated analysis aimed at revealing the genetic basis of obesity, MAGMA and POPS (30, 31) were used to identify and prioritize relevant genes, with *P*-values adjusted using the bonferroni procedure in each method. Genes with false discovery rate (FDR)-adjusted *P*-values of <0.05 and those consistently identified using both methods were considered significant risk factors. MAGMA enables gene-centered analysis based on extensive data from protein-coding genes and can be integrated with POPS to prioritize enriched genes. In particular, this approach integrates GWAS summary data with expression profiles and biological pathways, with a POPS score of >1 indicating candidate genetic risk factors.

To investigate the genetic correlates of obesity, SMR was performed using the GWAS summary data of patients with obesity and the eQTL data of various tissues and cell types (32). The inclusion criteria were as follows: FDR-adjusted *P*-value < 0.05; heterogeneity (HEIDI) > 0.01.

Phenotypic and genomic enrichment analyses were performed to assess the biological relevance of genes associated with obesity. Genomic enrichment analysis involved the use of data from the Molecular Signatures Database (MSigDB) (33), with significant biological pathways being identified using the ClusterProfiler tool after adjusting for multiple tests (34).

##### 2.2.4.3 Proteomic insights into obesity-depression comorbidity

The “Biomarker Level Inference from Summary Statistics” (BLISS) method was used to examine the complex proteomic landscapes of obesity and depression. Traditional proteome-wide association study (PWAS) models depend on detailed individual-level proteomic data. This dependence often limits the ability to utilize the vast amount of summary-level pQTL data available publicly (35). In contrast to traditional PWAS models, the BLISS method represents a novel strategy for constructing protein imputation models directly from summary-level pQTL data. In this study, the BLISS method was used to generate extensive European PWAS models using pQTL data from large-sample UKB, deCODE, and ARIC studies (35). Proteins with an FDR-adjusted P-value of <0.05 were identified as significant risk factors, indicating their potential key role in the pathophysiology of obesity and depression.

##### 2.2.4.4 Two-sample Mendelian randomization analysis

We conducted MR analysis to determine the causal link between obesity and depression (36, 37). Using the clumping procedure in PLINK software, we identified all significant genetic loci independently associated with these conditions (*P* < 5×10^-8^) as instrumental variables (IVs), with an r^2^ threshold of 0.001 and a window size of 10,000 kb. To ensure the robustness of our IVs, we calculated the coefficient of determination (r2) and the F-statistic, including only SNPs with an F-value greater than 10. The inverse-variance weighted (IVW) method was the primary analysis technique. We also conducted several sensitivity analyses by assessing heterogeneity among individual IVs using IVW and MR-Egger Q tests to identify potential violations of the assumptions. MR-Egger was further applied to account for horizontal pleiotropy based on its intercept estimate, ensuring that genetic variants were not related to both the exposure and the outcome. To strengthen the robustness of the results, we performed additional analyses using MR methods with different modeling assumptions and strengths, such as weighted median and weighted mode. Additionally, we used the MR-PRESSO method, repeated 1,000 times, to detect outliers (14), and any identified outliers were removed for reevaluation.

## 3 Results

### 3.1 Genetic relationship between obesity and depression

In our comprehensive investigation, we conducted LDSC analyses on GWAS data for obesity from two different sources and depression separately. Following stringent Bonferroni correction, we found significant genetic correlations between depression and obesity from both sources, which was further confirmed by HDL analysis. The results obtained through LDSC and HDL are presented in **Table 1**. We then meta-analyzed the GWAS summary statistics from the two sources of obesity, creating a combined dataset (META-OBESITY). This dataset includes 10,634,628 validated SNPs, meticulously excluding the complex MHC region, and identified 1,166 statistically significant genetic loci.

**Table 1:** Genetic correlation analysis results.

Trait pair	LDSC			HDL	
	rg (SE)	P	Intercept (SE)	rg (SE)	p
Obesity (FinnGen) - depression	0.208 (0.025)	2.92e-17	0.004 (0.006)	0.190(0.028)	1.68e-11
Obesity (UKbiobank) - depression	0.398 (0.053)	8.49e-14	0.010 (0.007)	0.330(0.037)	1.16e-18

### 3.2 Bidirectional two-sample MR

**Figure 2:**
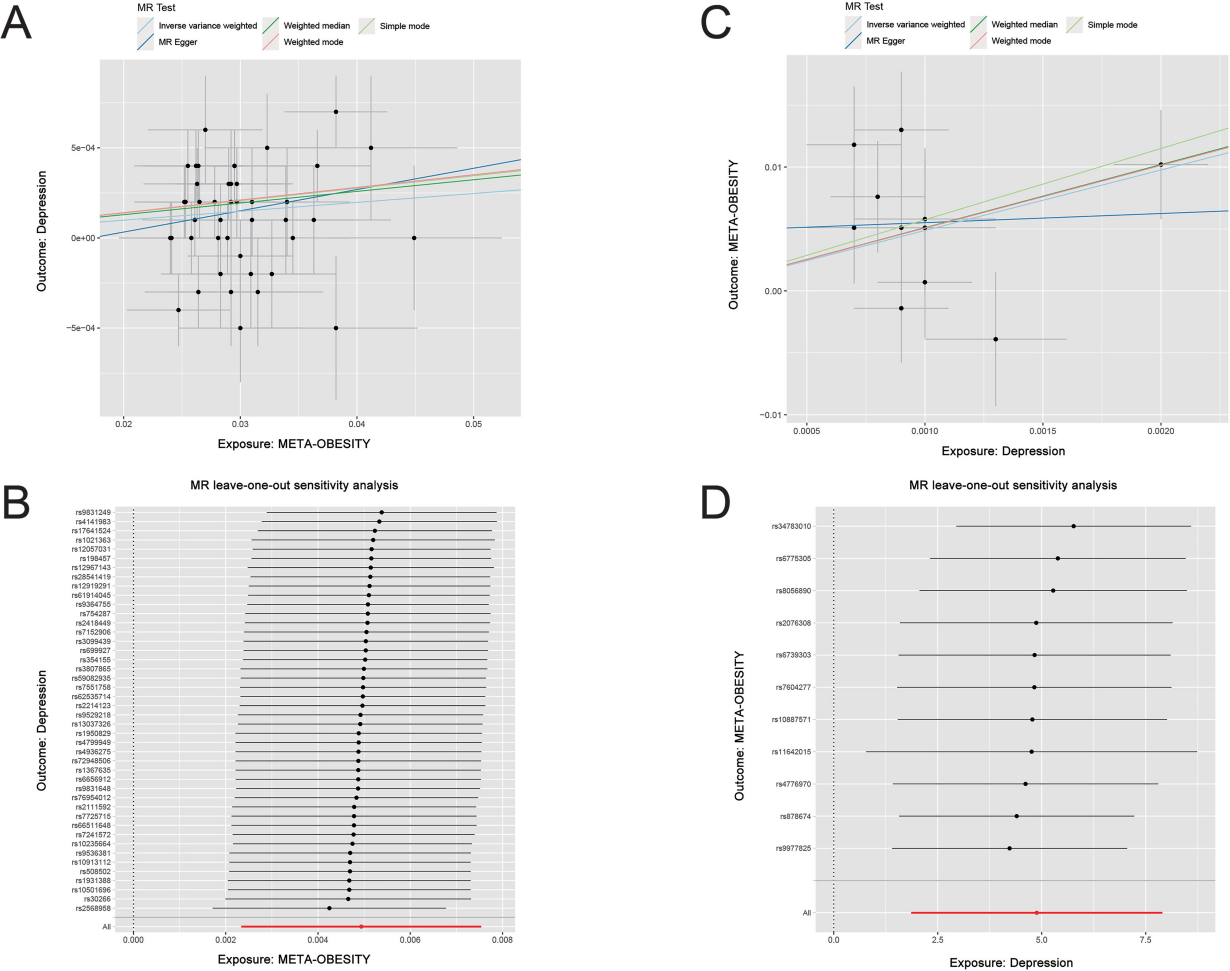
Scatter plot **(A)** and sensitivity analysis **(B)**, of the effect of META-OBESITY on depression. Scatter plot **(C)** and sensitivity analysis **(D)** of the effect of depression on META-OBESITY.

Our MR analysis indicated that obesity might increase the risk of depression (IVW, β=4.88, 95% confidence interval (CI): 1.86-7.90, P = 0.0015). Consistent results regarding the effect of obesity on depression were observed through MR-Egger causal estimates, weighted median, and weighted mode analyses (**Figure 2A**). In the reverse MR analysis, depression might increase the risk of obesity (IVW, β=0.0049, 95% CI: 0.0013-0.0002, P = 0.00019) (**Figure 2B**). Consistent directional effects of depression on obesity were observed through MR-Egger causal estimates, weighted median, and weighted mode analyses. In this bidirectional MR analysis, there was no evidence of significant horizontal pleiotropy or heterogeneity in Cochran’s Q test and MR-PRESSO test (**Supplementary Table S2**). Additionally, no potential outliers were identified among the selected IVs in the “leave-one-out” sensitivity test, demonstrating the robustness of the results (**Figures 2C, D**).

### 3.3 Deciphering the genetic architecture of obesity-depression comorbidity through multi-trait analysis

Our investigation further delved into the intricate genetic issues of obesity by conducting a multi-trait analysis using the combined dataset (META-OBESITY) and the GWAS summary data for depression phenotypes. Utilizing the MTAG method, we generated an enhanced dataset for obesity (MTAG-OBESITY), which includes 6,941,121 SNPs. Within this dataset, we identified 241 variant SNPs.

### 3.4 Identifying distinct genetic markers associated with obesity-depression comorbidity

Using the advanced GCTA-COJO tool for stepwise model selection, we conducted conditional and joint association analysis on the MTAG-OBESITY dataset. Through this rigorous process, we identified seven SNVs, as detailed in **Supplementary Table S3**. Subsequent identification using PLACO results on the FUMA platform revealed four additional SNVs, as shown in **Supplementary Table S4**. Notably, rs10789340 was consistently identified in both COJO and FUMA analyses, establishing it as an independent genetic risk locus for obesity-depression comorbidity. Further detailed gene annotation using the ANNOVAR tool highlighted that rs10789340, located intergenically in the RPL31P12 gene, was distinguished by a high CADD score of 12.37. This suggests a potential pathogenic role, as indicated in **Supplementary Table S4**.

### 3.5 Genes associated with the risk of obesity-depression comorbidity

**Figure 3:**
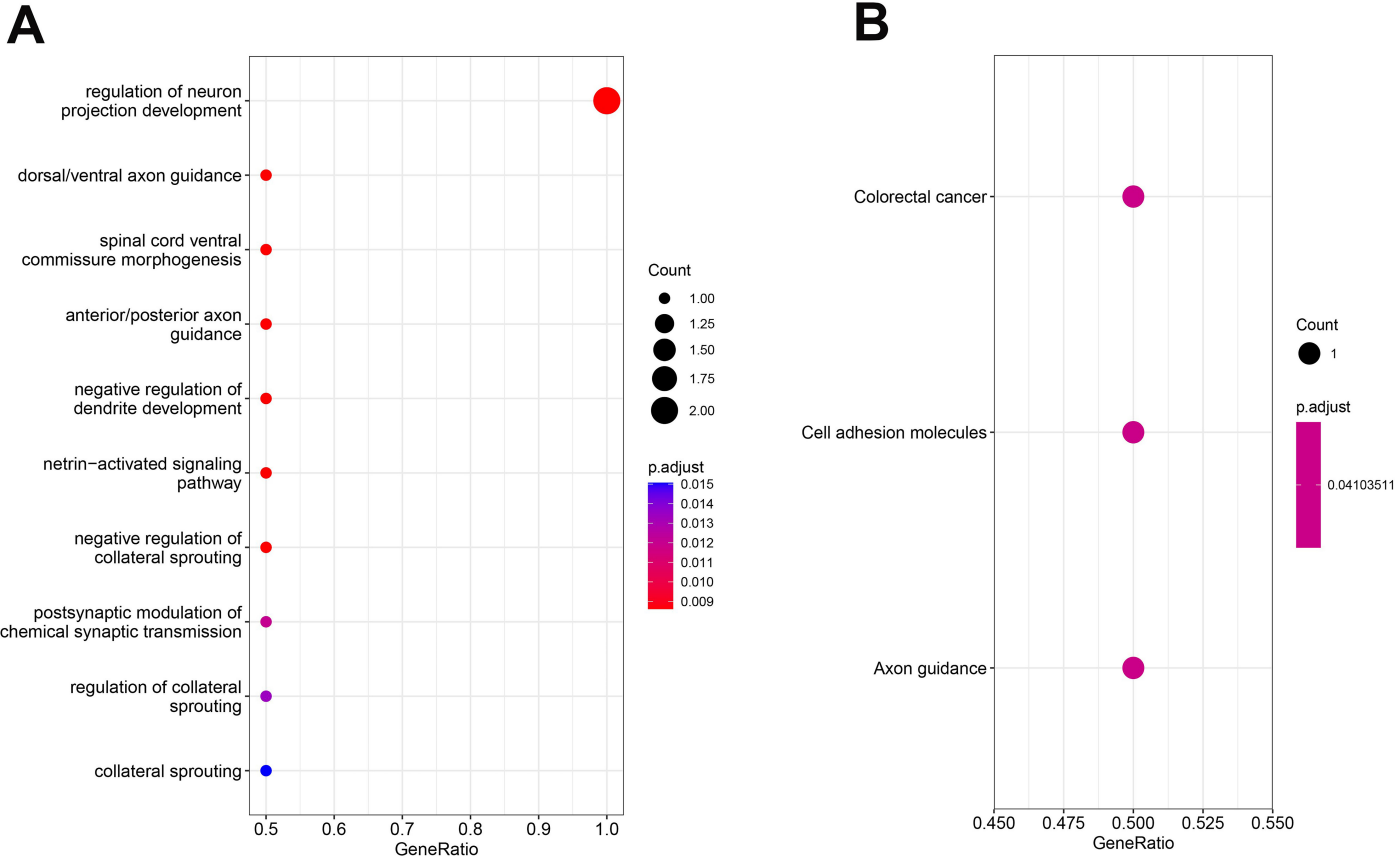
Enrichment analysis for identified risk genes. Significant Types of Pathways Based on GO **(A)** and KEGG Enrichment Analyses **(B)**. BP, Biological Process; CC, Cellular Component; MF, Molecular Function; KEGG, Kyoto encyclopedia of genes and genomes pathway.

MAGMA revealed 25 Genes associated with Obesity-Depression Comorbidity Risk SNVs (**Supplementary Table S5**). Subsequently, POPS revealed 1 gene (POPS scores > 1) potentially associated with the risk of Obesity-Depression Comorbidity(**Supplementary Table S6**). Additionally, SMR analysis identified RPL31P12 and NEGR1 as potential candidate genes (**Supplementary Table S7**). Interestingly, the SNP rs10789340 in RPL31P12 was also identified as a potential risk locus, further corroborating its role in obesity-depression comorbidity. Consequently, we identified three potential risk genes: DCC, RPL31P12, and NEGR1. Gene Ontology (GO) and KEGG pathway enrichment analyses indicated significant enrichment in pathways related to the regulation of nervous system development and feeding behavior (**Figures 3A, B**). These pathways include regulation of neuron projection development, neuron migration, axon guidance, and feeding behavior, suggesting that the intersection of obesity and depression comorbidity lies in the nervous system (**Supplementary Table S8**).

### 3.6 Identification of risk proteins associated with obesity-depression comorbidity

Through our analysis, we utilized the BLISS method in conjunction with the MTAG-OBESITY data to evaluate plasma proteins in the UKB, ARIC, and deCODE databases. This comprehensive analysis identified 56 proteins associated with the risk of obesity and depression comorbidity. Notably, two proteins, SCG3 and FLRT2, were consistently identified across all three databases (**Supplementary Table S9**), highlighting their potential as drug targets.

## 4 Discussion

The inherent complex genetic diversity in patients with obesity and comorbid depression necessitates moving beyond traditional single-disease frameworks to comprehensively explore the genetic foundations affecting human health and depression. This study aims to uncover the genetic basis and complex association network between obesity and depression.

Our analysis consolidated two obesity GWAS summaries into a single META-OBESITY dataset. Through genetic correlation studies of depression using LDSC and HDL, we found a significant genetic association between depression and obesity, further enhancing our understanding of obesity treatment. Integrating META-OBESITY with depression into comprehensive multivariate trait analysis aims to strengthen the statistical validation of the original obesity datasets. Additionally, bidirectional two-sample MR revealed a bidirectional positive causal relationship between obesity and depression. Using COJO and FUMA fine mapping techniques, we identified rs10789340 as an independent risk locus closely associated with comorbid obesity and depression. Notably, rs10789340 was not previously identified in individual obesity GWAS or META-OBESITY (*P* < 5×10^-8^). However, rs10789340 (*P* = 4.23×10^-17^) has been associated with depression in previous studies. This result highlights the significant role our study plays in identifying comorbid SNV, thereby deepening the understanding of the genetic basis of comorbid obesity and depression. Through this approach, we not only identified new genetic loci but also provided insights into the genetic diversity of obesity. Our comprehensive gene association analysis identified *DCC* as a gene associated with comorbid obesity and depression. Netrin-1, a protein encoded by *DCC*, contains three domains (38). Netrin-1 acts as a guidance cue, influencing the “decision-making” process of growing axons by signaling when, where, and whether to grow (39). It organizes neuronal circuits by attracting or repelling growing axons and dendrites, playing a crucial role in nervous system development, with its abnormal expression potentially leading to psychiatric disorders (40). A significant association between depression and epigenetic changes in *DCC* has been observed in individuals with major depressive disorder who died by suicide, showing a 50% increase in *DCC* gene expression in the prefrontal cortex of untreated patients (41). There is substantial genetic evidence from human populations indicating that variation in *DCC* expression is a risk factor for depression (42). Although these findings do not establish causality, animal studies using mouse models of depression have shown that increased *DCC* expression in the prefrontal cortex induces susceptibility to depression-like phenotypes (42). Recently, research by Ramkhelawon (43) has elucidated a pivotal function of netrin-1 in obesity, notably its role in anchoring adipose tissue macrophages within visceral adipose tissue. This mechanism substantially contributes to systemic inflammation and metabolic dysfunction. Further, a clinical investigation has revealed that levels of circulating netrin-1 are positively associated with critical markers such as fasting blood glucose, HbA1c levels, and the insulin resistance index. These findings imply that netrin-1 could serve as a sensitive biomarker for the early detection of type 2 diabetes (44), impacting glucose metabolism and indirectly exacerbating obesity. In summary, these studies collectively underscore netrin-1’s integral role in mediating obesity-related inflammation and its influence on the development of the nervous system. This knowledge brings to light the potential of *DCC* and its associated signaling pathways as prospective therapeutic targets for both obesity and depression.

**Figure 4:**
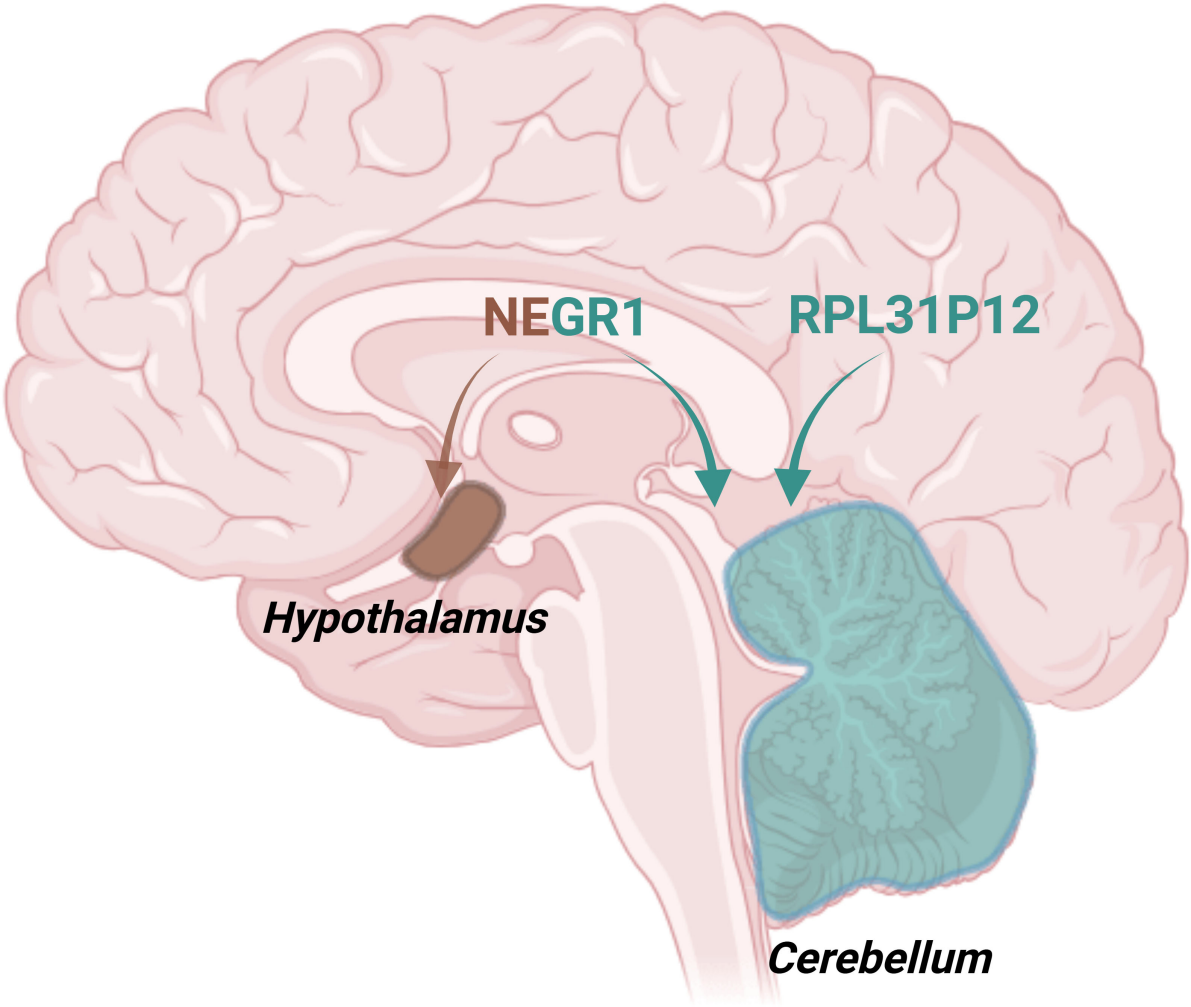
Schematic illustration of brain regions highlighting the hypothalamus and cerebellum. The brown area represents the hypothalamus, while the blue area corresponds to the cerebellum. NEGR1 is shown to primarily act in both the hypothalamus and cerebellum, while RPL31P12 predominantly influences the cerebellum. This figure was created using BioRender.

SMR analysis results identified *NEGR1* and *RPL31P12* as genetic links affecting the interaction between depression and obesity. *NEGR1* is thought to regulate cellular fat content by modulating the expression of CD36. In addition, *NEGR1* regulates body weight by influencing energy balance, lipogenesis, transport, and brain regions that control eating behavior, such as the hypothalamus and cerebellum (45, 46), highlighting its potential role in the pathogenesis of obesity. In human genetic studies, the rs10789336 in *NEGR1* was correlated with the expression levels of *RPL31P12* in brain tissue and an increased risk of major depressive disorder (47). *RPL31P12* exhibited a notably significant pleiotropic association with major depressive disorder, particularly in the cerebellum (48) (**Figure 4**). Additionally, diminished expression of a transcript variant of the *RPL31P12*, which encodes for the ribosomal protein L31 pseudogene 12, was linked to major depressive disorder (49), suggesting a shared genetic pathway influencing both depression and obesity risk.

Utilizing the BLISS method, we successfully identified 89 proteins implicated in the comorbid risk of obesity and depression across the deCODE, UKBPP, and ARIC databases, with *SCG3* and *FLRT2* detected in all three. *SCG3*, a notable member of the granin protein family (50), plays an essential role in endocrine and neuroendocrine cells, particularly in secretory and neurotransmitter vesicles through its interaction with chromogranin A (51). Previous research indicates that *SCG3* significantly influences glucose homeostasis by facilitating the formation of insulin-containing secretory granules and processing insulin (52), thereby impacting the body’s energy metabolism. Furthermore, cohort studies suggest that diminished *SCG3* levels are linked to an elevated risk of obesity (53). GWAS have pinpointed SNPs in the *SCG3* gene associated with obesity (54). Although direct evidence connecting *SCG3* to depression is absent, our findings propose a potential role for *SCG3* in the pathophysiology of depression.

Fibronectin leucine rich transmembrane protein (FLRT) family members are pivotal in cellular functions such as adhesion, migration, and axon guidance (55, 56). Specifically, FLRT2 has been shown to regulate dendritic spine density in CA1 neurons and to refine cortical circuits (57, 58) A proteomic analysis revealed notable differences in FLRT2 expression between the brains of hyperlipidemic and normal mice (59). Although no direct evidence currently connects FLRT2 to obesity or depression, its integral role in nervous system development underscores its potential as a therapeutic target for psychiatric disorders.

## 5 Limitation

This study, however, faces several limitations. The GWAS datasets utilized were derived from individuals of European descent, underscoring the necessity for additional research across more diverse populations to confirm the universality of our results. Additionally, the absence of individual-level GWAS data precluded the possibility of stratifying our analysis by age and sex, limiting our exploration of potential age- and sex-specific effects on the identified associations. Furthermore, the intrinsic constraints of GWAS, which primarily identify common genetic variants, often neglect rare or structural variants that could also contribute significantly to the comorbidity of obesity and depression.

## 6 Conclusion

Our study highlights the significant genetic association between obesity and depression, uncovering several novel genetic risk factors and associated biomolecules. These insights deepen our understanding of the genetic basis of the comorbidity between obesity and depression and may provide guidance for the development of new therapeutic strategies.

## Data Availability

The datasets presented in this study can be found in online repositories. The names of the repository/repositories and accession number(s) can be found in the article/**Supplementary Material**.
